# Paraneoplastic neurological syndrome with Guillain–Barré syndrome overlap in small-cell lung carcinoma: A case report from a primary care hospital in China

**DOI:** 10.1097/MD.0000000000047045

**Published:** 2026-01-09

**Authors:** Zhongyi Zhang, Xin Li, Deling Zhong

**Affiliations:** a Orthopedics Department,The First Affiliated Hospital of Zhejiang Chinese Medical University (Zhejiang Provincial Hospital of Chinese Medicine), Hangzhou City, Zhejiang Province, China; b Department of Neurology, Shengzhou Traditional Chinese Medicine Hospital, Shaoxing City, Zhejiang Province, China.

**Keywords:** anti-Hu antibody, case report, Guillain–Barré syndrome, paraneoplastic neurological syndrome, PET-CT, primary care, small cell lung carcinoma

## Abstract

**Rationale:**

The coexistence of paraneoplastic neurological syndrome (PNS) and Guillain–Barré syndrome (GBS) is an extremely rare clinical entity, and misdiagnosis is prevalent in grassroots healthcare settings due to limited diagnostic resources and atypical clinical presentations. Clarifying the diagnostic pathway for such overlap syndromes is critical to improving management in resource-limited primary care institutions.

**Patient concerns:**

A 75-year-old male presented with 4 months of persistent left facial numbness and 2 months of progressive distal limb numbness, accompanied by an unsteady gait and an unintentional 20 kg weight loss over 4 months. He was initially misdiagnosed with trigeminal neuritis and chronic inflammatory demyelinating polyneuropathy and had a history of hyperlipidemia managed with atorvastatin.

**Diagnoses:**

Serum anti-Hu antibody testing returned positive, while cerebrospinal fluid ganglioside antibodies were negative. Chest computed tomography identified mediastinal lymphadenopathy, and subsequent positron emission tomography-computed tomography revealed a hypermetabolic nodule in the right lower lung suggestive of primary malignancy. Bronchoscopic biopsy and immunohistochemistry confirmed small-cell lung carcinoma (SCLC). The final diagnosis was anti-Hu-positive PNS overlapping with GBS, secondary to SCLC, despite the absence of classic GBS clinical and laboratory features.

**Interventions:**

The patient received intravenous methylprednisolone pulse therapy (500 mg/d, tapered per clinical response), adjunctive gastrointestinal protection (pantoprazole), calcium/potassium supplementation, lipid-lowering therapy (atorvastatin), and neuroprotective agents (α-lipoic acid, methylcobalamin, vitamin B1). A multidisciplinary team coordinated neuro-oncological management following the SCLC diagnosis.

**Outcomes:**

During follow-up, the patient achieved a favorable recovery with significant alleviation of neurological symptoms, despite the SCLC’s high Ki-67 proliferation index (90%), attributed to early diagnostic confirmation and coordinated multidisciplinary care.

**Lessons:**

For patients with subacute neuropathy and unexplained weight loss in primary care settings, routine paraneoplastic antibody testing and whole-body imaging (e.g., positron emission tomography-computed tomography) are essential to facilitate early recognition of PNS–GBS overlap syndromes. Multidisciplinary collaboration across neurology, oncology, and radiology is key to timely management and improved prognosis in resource-limited healthcare environments, even in the absence of classic disease phenotypes.

## 1. Introduction

Paraneoplastic neurological syndrome (PNS) are rare, immune-mediated disorders triggered by tumor-associated antigens, most frequently affecting the central and peripheral nervous systems.^[1,2]^ Among these, anti-Hu antibody positivity is one of the most common serological markers, strongly associated with small cell lung carcinoma (SCLC).^[3–5]^ Although antibody testing can facilitate diagnosis and, in some cases, predict tumor type and prognosis,^[6]^ PNS remains underrecognized due to its heterogeneous manifestations. Approximately half of patients are diagnosed only after their malignancies reach advanced stages.^[7]^ Recent work continues to emphasize unresolved questions regarding the pathogenic role of anti-Hu antibodies and the mechanistic interplay between tumor biology and neurological injury.^[8–10]^

Guillain–Barré syndrome (GBS) is an immune-mediated peripheral neuropathy characterized by acute flaccid paralysis, with a global incidence of 0.6 to 4 per 100,000 and 0.698 per 100,000 in China.^[11]^ Despite therapeutic progress, substantial heterogeneity in clinical presentation and frequent residual sequelae pose ongoing challenges.^[12]^

The coexistence of PNS and GBS is exceptionally rare, complicating timely recognition and treatment. We report a case of anti-Hu-positive PNS overlapping with GBS, diagnosed in a primary care hospital in China. This case underscores the diagnostic value of antibody testing and neuroimaging in resource-limited settings and highlights the need for further research into the mechanisms, diagnostic strategies, and therapeutic approaches for such rare overlap syndromes.

## 2. Case report

A 75-year-old Chinese male presented with left facial numbness persisting for 4 months and distal limb numbness progressing over 2 months, culminating in unsteady gait. Initial evaluation during a prior hospitalization revealed no structural brain abnormalities on MRI/MRA. Diagnosed with trigeminal neuritis, he received dexamethasone (5 mg/day) with transient symptom relief, followed by traditional Chinese medicine. Despite this, neurological deficits worsened, with 20 kg unintentional weight loss over 4 months. Repeat MRA demonstrated atherosclerotic changes in cerebral arteries.

The patient was previously healthy except for hyperlipidemia diagnosed 2 months earlier, managed with atorvastatin calcium 20 mg nightly. There was no family history of neurological, infectious, or inherited disorders.

On examination, vital signs were stable (T 36.1°C, HR 77 bpm, RR 20/min, BP 104/64 mm Hg). The patient was alert and oriented, with normal cranial nerve function except for diminished light touch sensation over the left face. Motor strength was preserved (MRC grade 5 bilaterally), but coordination was mildly impaired on the left finger-to-nose test. Sensory deficits included reduced light touch in distal extremities and impaired vibration and proprioception in the lower limbs. Deep tendon reflexes were diffusely decreased, Romberg sign was positive, and Babinski signs were absent. No abnormalities were found in other systemic examinations.

**Figure 1.**
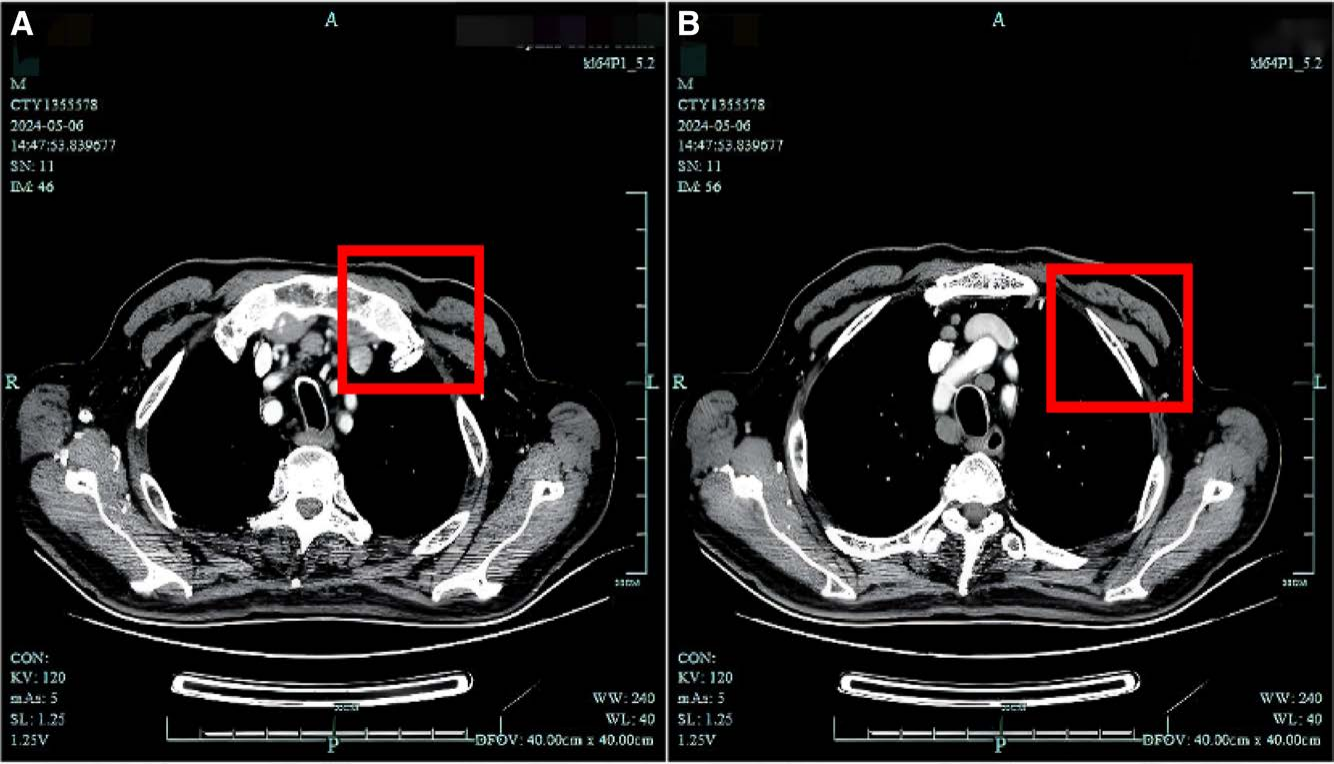
(A) and (B) Chest CT showing mediastinal lymph node abnormalities. CT = computed tomography.

Based on the clinical data and primary care protocols, we prioritized chronic inflammatory demyelinating polyradiculoneuropathy as the working diagnosis. Initial management included intravenous methylprednisolone pulse therapy (500 mg/day), tapered gradually based on clinical response. Adjunctive therapies comprised pantoprazole for gastrointestinal protection, calcium/potassium supplementation, atorvastatin for lipid control, and neuroprotective agents (α-lipoic acid, methylcobalamin, vitamin B1). As the patient presented with progressive neuropathy accompanied by significant weight loss, anti-Hu antibody testing was included as part of the extended work-up. Subsequent paraneoplastic antibody testing revealed serum anti-Hu antibody positivity, while cerebrospinal fluid ganglioside antibodies were negative. Chest CT identified multiple pulmonary nodules (Fig. 1A and B). Axial CT images demonstrate soft tissue density lesions in the mediastinal and paratracheal regions (highlighted by red rectangles), consistent with mediastinal lymphadenopathy.

**Figure 2.**
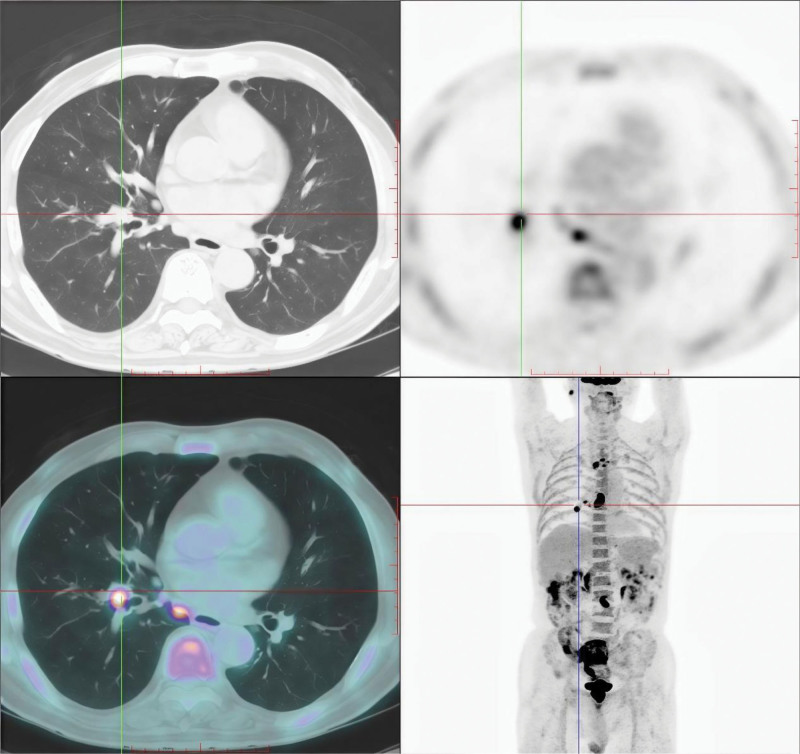
Metastatic lymphadenopathy with hypermetabolic activity in the mediastinum and right hilum PET-CT showing fluorodeoxyglucose-avid lesion in the right lower lung. PET-CT = positron emission tomography – computed tomography.

We initiated a systematic etiological evaluation for this complex case. Inflammatory, infectious, metabolic, and endocrine etiologies were excluded based on clinical history. The presence of rapid weight loss, progressive sensorimotor deficits, anti-Hu antibody positivity, and preferential sensory neuropathy – despite lacking overt tumor manifestations – strongly supported PNS, which often precedes tumor detection. However, persistent diagnostic uncertainty remained given the rarity of such presentations in primary care settings. PET-CT imaging revealed: FDG-avid solid nodule in the right lower lung dorsal segment, suggestive of primary malignancy (Fig. 2). These findings confirmed concomitant PNS and GBS, with radiological evidence of occult pulmonary malignancy.

**Figure 3.**
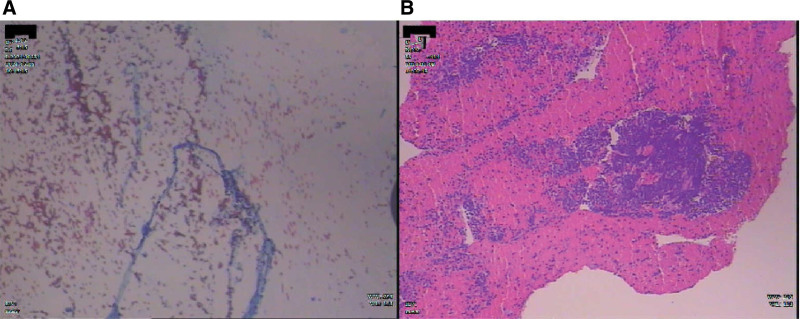
Histopathological examination of the lung (A) IHC staining demonstrating nuclear positivity in tumor cells. (B) H&E staining revealing typical morphological features of SCLC, including small-sized cells, hyperchromatic nuclei, scant cytoplasm, and mitotic figures. H&E = Hematoxylin and Eosin, IHC = immunohistochemical, SCLC = small cell lung carcinoma.

Definitive diagnosis was confirmed through bronchoscopic biopsy and immunohistochemistry (Fig. 3A and B), revealing SCLC with the following profile:LCA-negative, TTF-1-positive, NapsinA-negative, focal chromogranin A expression, synaptophysin-positive, CD56-positive, INSM1-positive, pan-cytokeratin-positive, P40-negative, and Ki-67 proliferation index of 90%. Our multidisciplinary team established the final diagnosis as PNS concurrent with GBS, mediated by remote neurological effects of SCLC.

**Figure 4.**
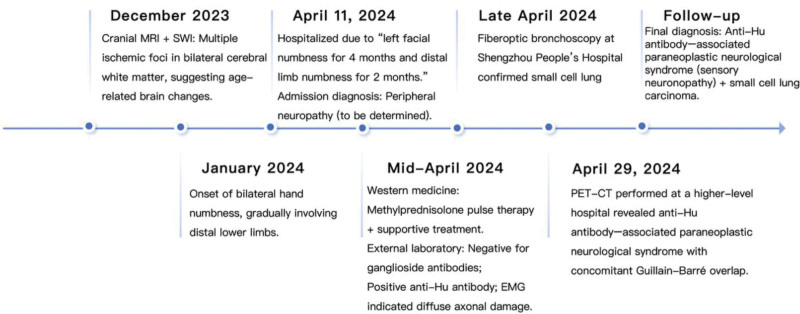
The timeline summarizes the clinical course from symptom onset to diagnostic work-up, pathological confirmation, and follow-up, highlighting key milestones in the diagnosis and management of PNS associated with small cell lung carcinoma. PNS = paraneoplastic neurological syndrome.

During follow-up (Fig. 4), the patient demonstrated favorable recovery despite the tumor’s high proliferative activity, attributable to early detection and coordinated neuro-oncological management, with significant alleviation of neurological symptoms.

## 3. Discussion

This case illustrates a rare overlapping syndrome of PNS and GBS, both secondary to SCLC. The patient’s initial misdiagnoses of trigeminal neuritis and chronic inflammatory demyelinating polyneuropathy highlight the diagnostic pitfalls of overlapping neuropathies. In China’s primary care hospitals, such challenges are amplified by limited access to advanced diagnostic modalities and reliance on conventional diagnostic paradigms, often delaying recognition.^[13,14]^ The progressive sensory neuropathy, profound weight loss, and delayed tumor detection in this patient exemplify the diagnostic complexities of these overlapping syndromes.

Serological and imaging examinations were crucial in overcoming diagnostic uncertainty. The detection of serum anti-Hu antibodies significantly altered the diagnostic trajectory. Anti-Hu antibodies are among the most common onconeural antibodies in SCLC-associated PNS, with positivity rates of 50% to 70%.^[5]^ Although absent in cerebrospinal fluid, this finding aligns with the concept of immunological compartmentalization in PNS. Early reports have shown that anti-Hu antibodies specifically target neuronal nuclear antigens and warrant thorough tumor evaluation.^[15]^ If tumors are not detected initially, they may become evident within months.^[16]^ In this case, subsequent PET-CT identified metabolically active pulmonary nodules and metastatic lymph nodes, and histopathology confirmed SCLC, consistent with evidence that up to half of PNS cases precede tumor diagnosis.^[17]^

The coexistence of PNS and GBS raises questions about shared immunopathogenic pathways. Anti-Hu antibodies may lead to neuronal injury in sensory ganglia and peripheral nerves, explaining the predominantly sensory deficits observed. At the same time, molecular mimicry between tumor antigens and peripheral nerve components could trigger GBS-like demyelination, as supported by reports of malignancy-associated GBS.^[18]^ However, the absence of cytoalbuminologic dissociation and ganglioside antibodies in this patient suggests a distinct paraneoplastic-driven immune phenotype, rather than classic GBS.

For clinicians in primary care hospitals, this case provides several lessons. Patients presenting with subacute neuropathy and unexplained weight loss should undergo antibody testing even in the absence of overt malignancy. PET-CT can be valuable for detecting occult tumors in seropositive PNS patients, although it is not yet routinely used in resource-limited settings.^[19]^ Effective management also requires multidisciplinary collaboration across neurology, oncology, and radiology to expedite diagnosis and treatment, which remains challenging in grassroots practice.^[16]^

This report has limitations. As a single case, causality between anti-Hu antibodies and peripheral nerve injury cannot be established with certainty. Treatment decisions were constrained by the availability of resources, limiting broader applicability. Nevertheless, this case underscores the importance of vigilance toward antibody-mediated neurological syndromes, demonstrates the diagnostic value of combining antibody detection and PET-CT in primary care hospitals, and highlights the clinical significance of recognizing such rare overlapping syndromes.

## 4. Conclusion

This case highlights the diagnostic and therapeutic complexities of rare PNS in China’s primary care settings, underscoring the need for heightened vigilance, systematic antibody testing, and early imaging screening in patients with atypical neuropathies. Future research should focus on unraveling the immunobiological mechanisms of antibody-mediated neural injury and optimizing multimodal treatment protocols to improve outcomes for this high-mortality population.

## Author contributions

**Conceptualization:** Zhongyi Zhang.

**Data curation:** Deling Zhong.

**Investigation:** Xin Li.

**Visualization:** Xin Li.

**Writing – original draft:** Zhongyi Zhang.

**Writing – review & editing:** Deling Zhong.
